# Severe neonatal Marfan syndrome with a novel mutation in the intron of the *FBN1* gene: A case report

**DOI:** 10.1097/MD.0000000000024301

**Published:** 2021-02-12

**Authors:** Su Hyun Yoon, Younghwa Kong

**Affiliations:** a Department of Pediatrics, Jeonbuk National University Hospital; b Research Institute of Clinical Medicine of Jeonbuk National University-Biomedical Research Institute of Jeonbuk National University Hospital, Jeonju, Republic of Korea.

**Keywords:** *fibrillin-1* gene, neonatal Marfan syndrome

## Abstract

**Rationale:**

Marfan syndrome (MFS) has been defined as a genetic disorder that affects various systems such as the musculoskeletal, orbital, and cardiovascular systems. Neonatal MFS is considered rare and the most severe form of MFS is characterized by rapidly progressive atrioventricular valve dysfunction, often leading to death during early childhood due to congestive heart failure.

**Patient concerns:**

A newborn with neonatal MFS and severe cardiac involvement. He presented various severe clinical features such as arachnodactyly, camptodactyly, elbow and knee joint contracture, senile facial appearance, and deep settling with down-slanting palpebral fissure, hypoplastic ear cartilage, sagging mouth, brachycephaly, and ectopia lentis.

**Diagnosis:**

Genetic analysis revealed a novel mutation at nucleotide 3964 (c.3964 + 1 G > T) in intron 32 of the *fibrillin-1* gene. This mutation is identified to be in the so-called neonatal region of *fibrillin-1* exon 24 to 32, as reported previously.

**Interventions:**

The patient was managed medically for improving the low cardiac output according to severe mitral regurgitation and aortic regurgitation. Afterload reduction, full sedation, and use of diuretic were attempted to improve the oliguria and heart failure.

**Outcomes:**

Despite the medical management, aortic regurgitation, mitral regurgitation, pulmonary hypertension, and cardiac contractility got worse. Surgical treatment is essential to prolong the patient's life, however, considerations for the grave progression of the disease make families decide to continue palliative care instead of surgical treatment. A few months after birth, he presented with rapidly progressive aortic regurgitation, mitral regurgitation, and congestive heart failure leading to death.

**Conclusions:**

This review demonstrated the prominent characteristics of neonatal MFS mutations, it would be helpful for the recognition of novel neonatal MFS variants and valuable for the understanding of the genotype-phenotype correlations and using the plans for managements and counseling in neonatal MFS.

## 1 Introduction

Marfan syndrome (MFS) is defined as a genetic disorder that affects various systems such as the musculoskeletal, orbital, and cardiovascular systems. Among the severe forms of MFS, neonatal MFS occurs early in life, mostly affecting the musculoskeletal system. Classic MFS shows clinical progression that gradually deteriorates with age, whereas neonatal MFS shows severe clinical manifestations and poor prognosis.^[1]^ This is associated with the progression of mitral and tricuspid valve regurgitation and aortic root dilatation caused by neonatal MFS, resulting in congestive heart failure. Recently, with the help of the development of diagnosis and treatment, it is has been reported that a good survival rate is possible even at 4 to 11 years of age, but the average survival age is 16.3 months, which is deemed very short.^[2,3]^ It is necessary to make a perinatal early diagnosis since the prognosis is determined according to the degree of cardiac involvement severity at the time of birth. In particular, a genetic diagnosis based on the international diagnostic criteria for MFS is preferred.^[4]^

Marfan syndrome is determined to occur at a frequency of 2 to 3 people per 10,000 people, regardless of gender or race, and is mainly caused by the mutation of *fibrillin-1* (*FBN1*).^[5]^*FBN1* mutations show a wide spectrum of phenotypes from neonatal to classic MFS as well as mild clinical symptoms. Meanwhile, it has been reported that neonatal MFS is associated with a small number of mutations in exons 24 to 32, the so-called neonatal region of the *FBN1* gene (MIM#134797).^[3]^

This study reported a case of neonatal MFS with severe congenital anomalies early in life, with a confirmed novel mutation, according to the genetic analysis, corresponding to intron 32, which is the neonatal region of the *FBN1* gene.

## 2 Case report

A male was born via an emergency cesarean section due to fetal distress at 40 weeks of gestational age. The mother's age was 33 years, with gravida 1 and para 1 parity. Both the parents and brother had no family history of congenital anomalies, aortic-related diseases, or sudden death. Based on the results of the prenatal ultrasonography at the end of the second trimester, the femur length of the fetus was found to be 1 to 3 weeks longer than the supposed length of the actual gestational age. Fetal echocardiography showed cardiomegaly with a fetal cardiothoracic circumference ratio of 0.5 or higher based on the baby's term. Moreover, the size of the foramen ovale was larger than normal, and left aortic constriction was seen next to the subclavian artery basin. Furthermore, no other abnormalities were found on prenatal ultrasound.

**Figure 1:**
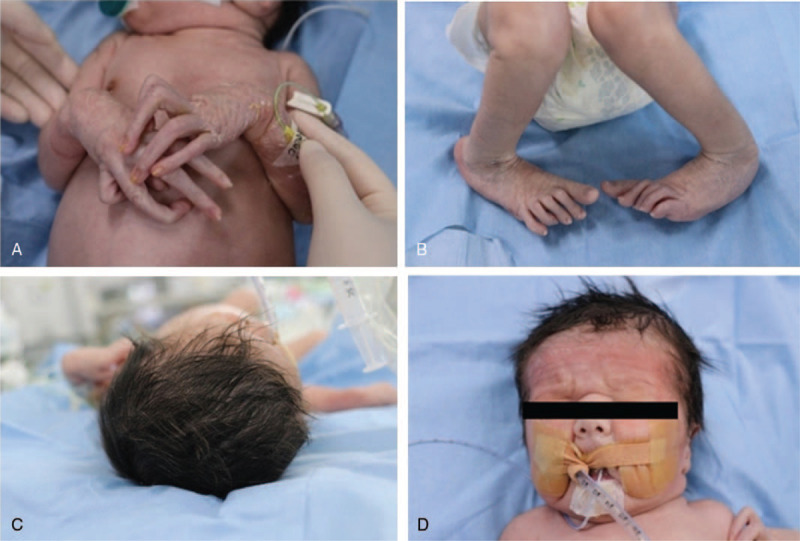
Congenital anomalies showing arachnodactyly of the hands (A) and feet (B), brachycephaly (C), and down-slanting palpebral fissure with senile facial appearance (D).

**Figure 2:**
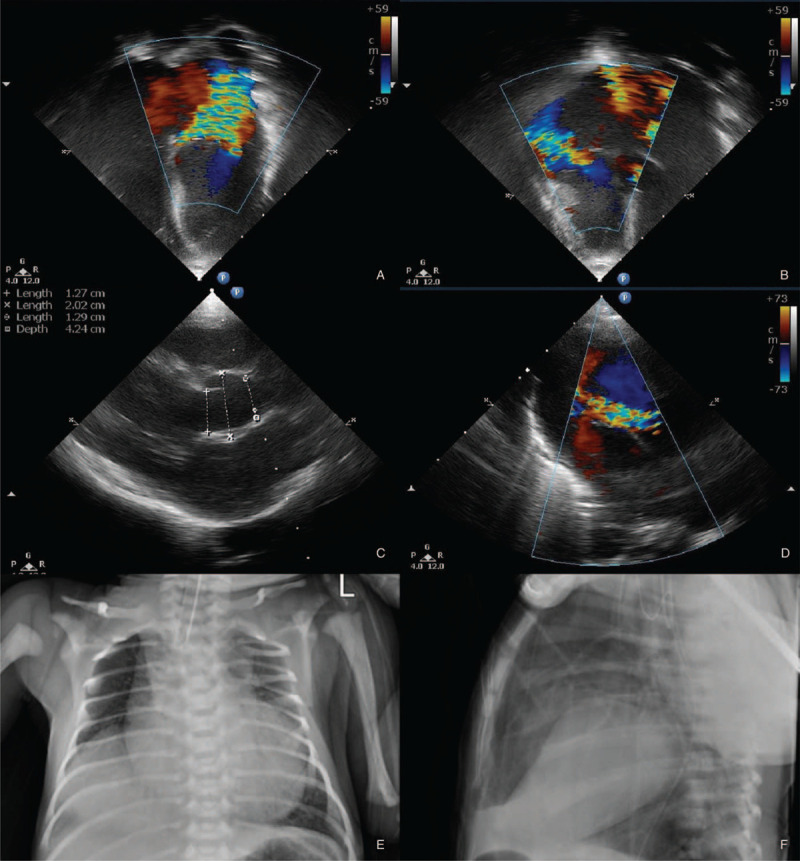
Echocardiography results showing severe mitral (A) and moderate tricuspid regurgitation (B) on 4-chamber views. Aortic root dilatation (C) on parasternal long axis view and moderate aortic regurgitation (D) on five-chamber view. X-ray suggesting liver herniation (C, D).

At birth, the weight was 3560 g (75 percentile), the length was 56.5 cm (over 90 percentile), and the head circumference was 36 cm (over 90 percentile). Apgar scores at 1 and 5 minutes were 4 and 6 points, respectively. In the delivery room, the patient had no spontaneous breathing and had bradycardia and cyanosis. After being admitted to the neonatal intensive care unit, various musculoskeletal malformations were confirmed via physical examination. Severe arachnodactyly and camptodactyly were observed in both hands and feet, and the soles of the feet were flat. The elbow and knee joints were not fully extended. The face had malar hypoplasia with senile facial appearance. The eye was deeply settled with a down-slanting palpebral fissure, and the ear with hypoplastic cartilage was poorly settled and crumpled. The patient presented with a sagging mouth, prominent coronal suture, and brachycephaly (Fig. 1). A grade V/VI systolic murmur was heard at both the upper sternal border and left lower sternal border with grade III parasternal heave. Echocardiography showed poor cardiac contractility, severe pulmonary hypertension, dilated aortic sinus (20.2 mm) (Z-score; 8.08 by Boston, 6.37 by Detroit, or 5.97 by Halifax), and multiple intracardiac valvular dysfunction with valve prolapses (moderate aortic regurgitation, severe mitral regurgitation, moderate tricuspid regurgitation, and moderate pulmonary valve regurgitation) (Fig. 2 A–D). And the ophthalmologic examination results showed ectopia lentis in both eyes as well as lens subluxation. Liver herniation was confirmed using abdominal X-ray and ultrasound (Fig. 2 E,F). The systemic score of the musculoskeletal manifestation was 11 points, according to the Ghent criteria (international diagnostic criteria for MFS).

**Figure 3:**
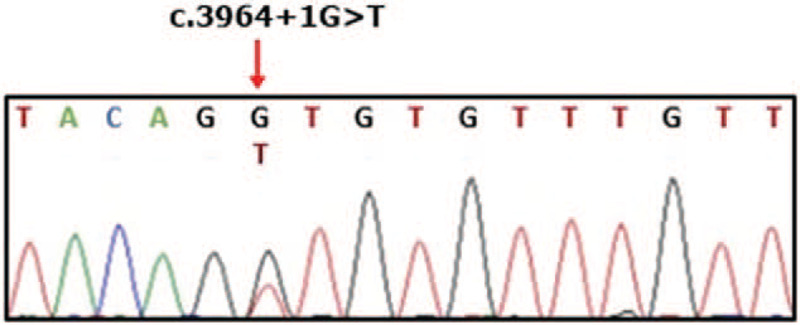
Sequence analysis of the region of the fibrillin-1 (FBN1) gene containing a mutation in which G, the first base of the 32nd intron in the form of heterogeneous mutation, is substituted with T (c.3964 + 1G > T).

For genetic diagnosis, Sanger sequencing and polymerase chain reaction were performed on the nucleotide sequence as reference for the *FBN1* gene. As a result, a mutation in which G, the first base of the 32nd intron in the form of a heterogeneous mutation, was substituted with T (c.3964 + 1G > T) (Fig. 3). This was confirmed as the likely pathogen variant based on the 2015 ACMG/AMP guideline. The location of the mutation was included in the site previously known as the neonatal region of MFS (exons 24–32). The patient could be diagnosed with neonatal MFS with a novel *FBN1* gene mutation within 2 weeks of life.

On the first day of life, differential cyanosis was found to show refractory hypoxemia despite more than 60% oxygen supply and signs of low cardiac output. The patient was managed medically for improving the low cardiac output according to severe mitral regurgitation and aortic regurgitation. Afterload reduction including milrinone continuous infusion, full sedation using fentanyl continuous infusion, and use of diuretic were attempt to improve the oliguria and heart failure. Despite the medical management, the patient presented with respiratory failure, heart failure, and severe pulmonary hypertension requiring continuous invasive mechanical ventilation. Aortic regurgitation, mitral regurgitation, pulmonary hypertension, and cardiac contractility got worse. After several consultations with the patient's family and medical staff about the treatment plan, palliative care was continued instead of surgical treatment. As a result, hepatic and pulmonary congestion accompanied by pulmonary hemorrhage had progressed as well. Eventually, it had progressed to multiple organ dysfunction syndrome, and the patient died 32 days after the birth.

## 3 Discussion

This study reported a case of neonatal MFS with a novel mutation (c.3964 + 1G > T) in the intron 32nd region of *FBN1*. Moreover, 2 reported cases in which the same position is substituted with a different base sequence are found, and they have been diagnosed with severe neonatal MFS.^[6–8]^ In accordance with previously reported cases, this patient was diagnosed with neonatal MFS from the beginning of life, with severe cardiac congenital anomalies, and showed poor prognosis leading to death.^[7]^

The *FBN1* gene of chromosome 15 (; MIM#134797) has been identified as a major component of microfibrils and is involved in the formation of the extracellular matrix or elastin fibers.^[8]^ In particular, the region of exon 24 to 32, known as the “neonatal region,” is about 5% of the *FBN1* gene, showing severe and various phenotypes related to neonatal MFS.^[7,9]^ Previous studies have shown that some mutations in this region may cause a loss of function for heparin binding and enhanced proteolytic susceptibility of *FBN1*, which resulted in more severe phenotypes of MFS.^[10]^ Although patients with a mutation in the site known as neonatal MFS are often accompanied by other malformations with typical MFS early in life, the genotype-phenotype correlation for MFS is yet to be investigated further. Moreover, considering that neonatal MFS has a severe form of accompanying malformations different from classic MFS, it is necessary to have diagnostic criteria for neonatal MFS.^[1,4]^ Echocardiography of this patient early in life has shown a severe form of mitral regurgitation, which can also be observed in previously reported cases of neonatal MFS. In addition, he presented with multiple musculoskeletal anomalies with valvular heart disease, which is also consistent with the previous cases of neonatal MFS. These factors could be important clues for the diagnosis of neonatal MFS in the early stages of life.

The main cause of death in typical MFS is the rupture of an ascending aortic aneurysm, which in neonatal MFS is due to congestive heart failure secondary to mitral regurgitation. In addition, neonatal MFS has a very poor prognosis; approximately 50% of patients develop congestive heart failure within 1 year of life. Therefore, it is very important to make treatment plans and predict the progression of the disease in the early stages of life. Since valvular failure and aortic dissection are the most serious clinical manifestations of neonatal MFS, medical treatment alone cannot prevent its progression to congestive heart failure or sudden death. According to a recent study, surgical treatment is known to increase the survival rate of patients with neonatal MFS, and surgical treatment must be considered in a timely manner.^[2,11,12]^ Therefore, proper diagnosis and treatment of neonatal MFS are necessary during the perinatal period.

However, the genotype-phenotype correlation due to the mutation in the neonatal region has not yet been sufficiently investigated. The database is gradually secured, and the prognosis according to the genotype will be revealed.^[9]^ Because neonatal MFS shows a grave process from birth to the rapid progression of congestive heart failure leading to death with unstable vital signs, it is necessary to provide a basis for predicting the phenotype.

In conclusion, we believe that this study on neonatal MFS with a novel mutation of the *FNB1* gene may be helpful in other studies focusing on cases with mutations in the neonatal region. Furthermore, this will also be valuable in future studies on the genotype-phenotype correlation for neonatal MFS.

## Acknowledgment

The authors thank the family for their support in reporting this case study for providing consent to take the medical data and clinical photographs of their son.

## Author contributions

**Conceptualization:** Su Hyun Yoon, Younghwa Kong.

**Investigation:** Su Hyun Yoon.

**Supervision:** Younghwa Kong.

**Writing – original draft:** Su Hyun Yoon.

**Writing – review & editing:** Younghwa Kong.

**Final approval of the manuscript:** Younghwa kong.

## Abbreviations

Abbreviations: *FBN1* = fibrillin-1, MFS = Marfan syndrome.
